# Association between organophosphorus pesticides and obesity among American adults

**DOI:** 10.1186/s12940-024-01104-z

**Published:** 2024-07-20

**Authors:** Wei Xu, Yinqiao Dong, Shiping Liu, Fan Hu, Yong Cai

**Affiliations:** 1 Department of Emergency, Ruijin Hospital, Shanghai Jiao Tong University School of Medicine, Shanghai, 200025 China; 2 Department of Public Health, Tongren Hospital, Shanghai Jiao Tong University School of Medicine, Shanghai, 200335 China; 3 School of public health, Shanghai Jiao Tong University School of Medicine, Shanghai, 200025 China; 4 National Children’s Medical Center, Shanghai Children’s Medical Center affiliated to Shanghai Jiao Tong University School of Medicine, Shanghai, 200127 China

**Keywords:** Organophosphate pesticides, Obesity, NHANES, WQS, Qgcomp

## Abstract

**Objective:**

To investigate any connections between urinary organophosphorus **pesticide** (OPP) metabolites and adiposity measures.

**Methods:**

In this study, data from the National Health and Nutrition Examination Survey (NHANES) projects from 2003 to 2008, 2011 to 2012, and 2015 to 2018 were analysed. Obesity was defined as a body mass index (BMI) of 30 kg/m² or higher. Abdominal obesity was defined as a waist circumference (WC) over 102 cm for men and 88 cm for women. Four urinary OPP metabolites (dimethyl phosphate [DMP], diethyl phosphate [DEP], dimethyl phosphorothioate [DMTP], and diethyl phosphorothioate [DETP]) and adiposity measures were examined using multiple linear regression and logistic regression analyses. The correlations between a variety of urinary OPP metabolites and the prevalence of obesity were investigated using weighted quantile sum regression and quantile g-computation regression.

**Results:**

In this analysis, a total of 9,505 adults were taken into account. There were 49.81% of male participants, and the average age was 46.00 years old. The median BMI and WC of the subjects were 27.70 kg/m^2^ and 97.10 cm, respectively. Moreover, 35.60% of the participants were obese, and 54.42% had abdominal obesity. DMP, DMTP, and DETP were discovered to have a negative correlation with WC and BMI in the adjusted models. DMP (OR = 0.93 [95% CI: 0.89–0.98]), DEP (OR = 0.94 [95% CI: 0.90–0.99]), DMTP (OR = 0.91 [95% CI: 0.86–0.95]), and DETP (OR = 0.85 [95% CI: 0.80–0.90]) exhibited negative associations with obesity prevalence. Similar correlations between the prevalence of abdominal obesity and the urine OPP metabolites were discovered. Moreover, the mixture of urinary OPP metabolites showed negative associations with adiposity measures, with DMTP and DETP showing the most significant effects.

**Conclusion:**

Together, higher levels of urinary OPP metabolites in the urine were linked to a decline in the prevalence of obesity.

**Supplementary Information:**

The online version contains supplementary material available at 10.1186/s12940-024-01104-z.

## Introduction

Obesity incidence is quickly rising worldwide, placing a significant burden on both individuals and society [1, 2]. Two-thirds of all American adults are overweight, which has significant health consequences [3, 4]. Waist circumference (WC) is a sign of visceral fat and a stronger predictor of obesity-related health hazards, even though present research mainly focuses on body mass index (BMI) to define obesity trends [5]. Furthermore, chemical exposure is getting increasing attention for its potential roles in obesity compared to factors like dietary practices, lifestyle, genetics, race, and socioeconomic position [6].

Human exposure to organophosphorus pesticides (OPPs) is prevalent since they are among the most widely used pesticides [7], including Chlorpyrifos, Bensulide, and Acephate, with chlorpyrifos being banned in 2021 [8]. OPPs can be ingested, inhaled, or applied directly to the skin and cause a variety of metabolites [9], including dimethyl phosphate (DMP), dimethyl phosphorothioate (DMTP), dimethyl dithiophosphate (DMDTP), diethyl phosphate (DEP), diethyl phosphorothioate (DETP), and diethyl dithiophosphate (DEDTP) [10]. The metabolic process is then carried out by the pesticides in two stages [11]. The metabolic enzymes first reduce, oxidize, or hydrolyze the pesticide chemicals into more water-soluble molecules. Next, the water solubility is increased for excretion in urine by conjugation with hydrophilic molecules. Therefore, in environmental and occupational research, urinary OPP metabolites are used as indicators of OPPs exposure [12].

Available evidence suggests that OPPs and their environmental residues adversely affect human health. Acute exposure to high doses of OPPs can have serious adverse effects on multiple systems, including cardiovascular, respiratory, endocrine, and neurological [13–15]. Diseases are linked to long-term exposure to low concentrations of OPPs [15], including Parkinson’s disease [16], breast cancer [17], diabetes [18] and liver damage [19]. A link between OPPs exposure and weight growth has been observed in earlier research [20]. According to a study conducted on family farmers in southern Brazil, chronic usage of OPPs is linked to a higher frequency of overweight people [21]. Eight OPPs were among the twenty-two pesticide exposures that were significantly linked to a higher frequency of obesity, according to a study from Thailand [22]. However, these studies have concentrated on people who have significant occupational OPPs exposure, and it is still unknown whether obesity is linked to levels of OPPs exposure in the general population. In addition, opposite results were observed in the NESCAV study, where polychlorinated biphenyls (PCBs), pentachlorophenol in hair were negatively associated with obesity [23]. Related studies showed that exposing mice to a variety of OPPs with similar toxicity mechanisms caused body weight restriction in vivo. Potential toxicity mechanisms included DNA damage, oxidative stress, liver and renal dysfunction, alterations in lipid metabolism, and increased protein and lipid degradation [24]. There is a lot of conflicting evidence in the literature about how OPPs exposure affects body weight. Thus, this study used data from a sizable representative population to examine the relationships between four specific urine OPP metabolites and adiposity measures in American adults.

## Materials and methods

### Study population

A significant population-based study that evaluates the health and nutritional status of both children and adults is the National Health and Nutrition Examination Survey (NHANES). Since 1999, it has been gathering participant interview and physical examination data during a two-year cycle. The official NHANES website (https://www.cdc.gov/nchs/nhanes/index.htm) allows users to download all NHANES data. The investigation was authorized by the Research Ethics Review Board of the Centers for Disease Control and Prevention, and each participant was informed of the details and signed the informed consent form before the study began.

Since the cycles containing urinary OPP metabolites data were 2003–2004, 2005–2006, 2007–2008, 2011–2012, 2015–2016, and 2017–2018. So, we use six survey cycles to conduct this study. In total, we initially enrolled 59,600 participants from the six investigative cycles described above, and 44,224 subjects were removed because they lacked urine OPP metabolite-related data. Participants aged < 18 years (*n* = 4,975) and those with missing BMI and WC data (*n* = 658) were also excluded. Pregnant women were also excluded (*n* = 238). Finally, this study had 9,505 participants in total (Figure S1).

### Assessment of urinary OPP metabolites

The National Center for Environmental Health at the Centers for Disease Control and Prevention in Atlanta, Georgia, received the processed, stored, and transported urine samples from the participants for analysis. The concentrations of six OPP metabolites (DMP, DEP, DMTP, DETP, DMDTP and DEDTP) in urine were determined by ultrahigh-performance liquid chromatography-tandem mass spectrometry (UPLC-MS) based on solid phase extraction combined with isotope dilution pre-treatment [25]. The lower limit of detection (LOD) for all six OPP metabolites in urine was 0.1 ng/mL. The concentration and distribution of each of these urinary OPP metabolites were analysed. The detection rates of DMP, DEP, DMTP, DETP, DMDTP and DEDTP were 71.1%, 69.9%, 83.7%, 52.5%, 40.6% and 3.5%, respectively. Owing to the low detection rates of DMDTP and DEDTP, we only analysed four urinary OPP metabolites (DEP, DMP, DETP and DMTP). The real LOD was divided by $\:\sqrt{2}$ if the measured detection rates fell below the limit of detection.

### Assessment of adiposity measures

The anthropometric measurements of the subjects, including their height (m), weight (kg), and WC (cm), were taken using accepted practices. The kilograms per square meter (kg/m^2^) ratio of weight to height was used to compute BMI. BMI ≥ 30 kg/m^2^ was considered as obesity status [26]. A WC of more than 102 cm for men and more than 88 cm for women was deemed to be abdominal obesity [27].

### Covariates

Baseline information on participants was gathered from questionnaires and laboratory test results, and included their gender (female or male), age (years), race/ethnicity (other Hispanic, Mexican American, non-Hispanic white, non-Hispanic black, or other race), education level (< high school, high school, or > high school), and urinary creatinine level (mg/dL). Poverty is defined as a particular household with a poverty income ratio below 1 [28]. Smoking status was categorized into three groups: never smokers, former smokers, and current smokers. Non-drinker, low-to-moderate drinker, and heavy drinker were the three categories used to classify the drinking status. Based on previous evidence, physical activity in this study was classified into three groups: inactive (no leisure-time physical activity), insufficiently active (moderate or vigorous activity), and active (greater than moderate or vigorous activity) [29, 30]. Self-reported questionnaires were used to gather data on the prevalence of diabetes and hypertension. For comprehensive definitions of smoking status, drinking status, physical activity, healthy eating index (HEI), and total energy intakes, please refer to the methodology section of the supplementary materials.

### Statistical analysis

To provide accurate national estimates, sample weights, strata, and primary sampling units were taken into account in the data analysis. Weighed analyses were performed utilizing the “survey” R package. Medians (interquartile range) were used to present continuous variables. Categorical variables were displayed as percentages of numbers. In order to normalize the distributions of the urine OPP metabolites, log transformation was used. The correlation coefficients between all of the urine OPP metabolites were evaluated using Spearman correlation analysis. R software (version 4.2.0) was used for all statistical analyses, and a two-sided *P* value of 0.05 was regarded as statistically significant.

The four urine OPP metabolites linked to BMI and WC were assessed using the adjusted-coefficients and 95% confidence intervals (CIs) derived from multiple linear regression models. The four urine OPP metabolites were assessed for their associations with the prevalence of obesity and abdominal obesity using multiple logistic regression models, which were used to construct adjusted odds ratios (ORs) and 95% confidence intervals (CIs).

Weighted quantile sum (WQS) regression was carried out using R software package (“gWQS”) to evaluate the combined effects of numerous exposure variables on a given outcome in light of the substantial correlations among the urinary OPP metabolites. We evaluated the relationship between the combination of urine OPP metabolites and outcomes associated with obesity using this model. A weight was given to each exposure variable in the model, indicating how much of an impact it would have on the result. With a total of 2000 bootstrap samplings, we constructed the WQS index for adiposity measures based on OPP metabolites concentration deciles and used 40% of the data as a test set and 60% as a validation set.

To overcome the limited assumptions of the WQS method, we used the quantile g-computation (Q-gcomp) model to evaluate the mixture effects of urinary OPP metabolites with different contribution index directions. This complementary approach adequately integrates the advantages of the flexibility of g-calculation and the simplicity of inference of weighted quantile regression (WQS) to provide unbiased extrapolations of the combined effects of urine OPP metabolites mixtures [31]. Since the weights in the Q-gcomp analysis are flexible and can go either way, it is possible that some exposures could be advantageous and others potentially damaging [32]. The positive and negative weight coefficients for each component of the mixture were obtained using the “qgcomp” package.

## Results

### Baseline patient characteristics

The baseline characteristics of the 9,505 participants in the final analysis are shown in Table 1. The median participant age was 46.00 (32.00, 59.00) years, and 49.81% of the participants were men. The proportion of non-Hispanic whites was 40.47%. The median BMI and WC of the participants was 27.70 (24.10, 31.99) kg/m^2^ and 97.10 (86.40, 107.90) cm, respectively. The prevalence of obesity and abdominal obesity in adult participants was 35.60% and 54.42%, respectively. Participants in the obese group were older, less educated, less physically active, and more likely to have diabetes and hypertension than those in the non-obese group. In addition, there was no difference in the level of dietary energy intake between these two groups.

**Table 1:** Survey-weighted characteristics of adult NHANES 2003–2008, 2011–2012, and 2015–2018 participants with available data on urinary organophosphate metabolites

Characteristics	Total (*n* = 9505)	Obesity		*P* value
		No (*n* = 5998)	Yes (*n* = 3507)	
Age, years	46.00 (32.00, 59.00)	45.00 (30.00, 59.00)	47.00 (35.00, 60.00)	< 0.001
Male, %	4734 (49.81)	3155 (49.83)	1579 (47.54)	0.110
Race/ethnicity, %				< 0.001
Mexican American	1620 (17.04)	966 (7.65)	654 (10.08)	
Other Hispanic	836 (8.8)	519 (5.64)	317 (5.72)	
Non-Hispanic White	3847 (40.47)	2545 (68.61)	1302 (63.06)	
Non-Hispanic Black	2169 (22.82)	1157 (9.23)	1012 (14.94)	
Other race	1033 (10.87)	811 (8.88)	222 (6.19)	
Education level, %				0.002
Below high school	2420 (25.46)	1515 (15.35)	905 (16.40)	
High school	2163 (22.76)	1311 (22.34)	852 (25.82)	
Above high school	4922 (51.78)	3172 (62.31)	1750 (57.78)	
Poverty, %	2069 (21.77)	1290 (13.48)	779 (14.46)	0.250
Smoking status, %				0.003
Never smoker	5320 (55.97)	3378 (56.06)	1942 (55.28)	
Former smoker	2172 (22.85)	1283 (21.76)	889 (25.62)	
Current smoker	2013 (21.18)	1337 (22.18)	676 (19.10)	
Drinking status, %				0.030
Nondrinker	2254 (23.71)	1354 (18.15)	900 (19.79)	
Low-to-moderate drinker	6517 (68.56)	4131 (71.79)	2386 (72.41)	
Heavy drinker	734 (7.72)	513 (10.06)	221 (7.80)	
Physical activity, %				< 0.001
Inactive	2363 (24.86)	1377 (17.84)	986 (23.06)	
Insufficiently active	3436 (36.15)	2127 (38.24)	1309 (40.60)	
Active	3706 (38.99)	2494 (43.92)	1212 (36.34)	
Energy intake, kcal/day	2007.50 (1539.00, 2557.00)	1997.00 (1545.50, 2549.50)	2023.00 (1533.00, 2571.50)	0.870
Urinary creatinine, mg/dL	111.00 (63.00, 169.00)	104.00 (56.00, 164.00)	123.00 (76.00, 179.00)	< 0.001
Hypertension, %	3201 (33.68)	1592 (23.33)	1609 (42.44)	< 0.001
Diabetes, %	1123 (11.81)	510 (5.44)	613 (14.21)	< 0.001
Body mass index, kg/m^2^	27.70 (24.10, 31.99)	25.10 (22.60, 27.50)	33.88 (31.60, 37.89)	< 0.001
Waist circumference, cm	97.10 (86.40, 107.90)	90.00 (81.80, 97.30)	112.10 (105.30, 120.80)	< 0.001
Abdominal obesity, %	5173 (54.42)	1815 (31.36)	3358 (96.00)	< 0.001

Data are presented as median (interquartile range [IQR]) or *n* (%); Sampling weights were applied for calculation of demographic descriptive statistics; *N* reflect the study sample while percentages reflect the survey-weighted

### Distribution of and correlation among the urinary OPP metabolites

Table S1 presents the concentrations and detection rates of the urinary OPP metabolites. Metabolites with a detection rate of > 50% were considered. The final analysis comprised four OPP metabolites, including DMP, DEP, DMTP, and DETP. Furthermore, the results in Table S2 demonstrate statistically significant trends over time in the estimated weighted and geometric means of urinary OPP metabolites, with varying concentrations observed across different NHANES cycles. Figure S2 presents the Spearman correlation coefficients among the four urinary OPP metabolites. A strong correlation was observed between DMTP and DETP (*r* = 0.50). Moreover, strong correlations were also observed among DMP, DEP and DMTP (*r* = 0.42 and 0.49).

### Association between the urinary OPP metabolites and BMI and WC

Table 2 summarizes the outcomes of the statistical analysis performed by linear regression methods. DMP, DMTP and DETP were negatively correlated with the BMI after adjusting for urinary creatinine levels (all *P* < 0.001). This relationship also existed in model 2. In the full-adjusted model, the β coefficient (95% CI) of the BMI was − 0.30 (− 0.41, − 0.18) for DMP, − 0.22 (− 0.33, − 0.10) for DEP, − 0.37 (− 0.48, − 0.26) for DMTP and − 0.59 (− 0.75, − 0.43) for DETP. Moreover, DMP, DEP, DMTP and DETP were negatively correlated with the WC. The adjusted β coefficients (95% CI) of WC were − 0.75 (− 1.04, − 0.46) for DMP, − 0.54 (− 0.81, − 0.28) for DEP, − 0.92 (− 1.19, − 0.65) for DMTP and − 1.34 (− 1.72, − 0.95) for DETP in model 3.

**Table 2:** Multiple linear regression associations of urinary organophosphorus pesticide (OPP) metabolites with BMI and waist circumference in adults

OPs	Model	BMI, kg/m^2^		Waist circumference, cm	
		β (95%CI)	*P* value	β (95%CI)	*P* value
DMP	Model 1	-0.24 (-0.36, -0.12)	< 0.001	-0.60 (-0.92, -0.28)	< 0.001
	Model 2	-0.32 (-0.44, -0.19)	< 0.001	-0.80 (-1.11, -0.49)	< 0.001
	Model 3	-0.30 (-0.41, -0.18)	< 0.001	-0.75 (-1.04, -0.46)	< 0.001
DEP	Model 1	-0.06 (-0.18, 0.06)	0.304	-0.17 (-0.46, 0.13)	0.264
	Model 2	-0.13 (-0.25, -0.01)	0.034	-0.32 (-0.58, -0.05)	0.021
	Model 3	-0.22 (-0.33, -0.10)	< 0.001	-0.54 (-0.81, -0.28)	< 0.001
DMTP	Model 1	-0.40 (-0.52, -0.27)	< 0.001	-0.91 (-1.22, -0.59)	< 0.001
	Model 2	-0.46 (-0.59, -0.33)	< 0.001	-1.18 (-1.49, -0.86)	< 0.001
	Model 3	-0.37 (-0.48, -0.26)	< 0.001	-0.92 (-1.19, -0.65)	< 0.001
DETP	Model 1	-0.62 (-0.79, -0.44)	< 0.001	-1.44 (-1.91, -0.98)	< 0.001
	Model 2	-0.68 (-0.85, -0.50)	< 0.001	-1.63 (-2.06, -1.19)	< 0.001
	Model 3	-0.59 (-0.75, -0.43)	< 0.001	-1.34 (-1.72, -0.95)	< 0.001

BMI, body mass index; CI, confidence interval

Model 1 was adjusted for urinary creatinine;

Model 2 was adjusted for age, sex, race, and urinary creatinine;

Model 3 was adjusted for age, sex, race, urinary creatinine, poverty, smoking status, drinking status, energy intake levels, HEI, physical activity, diabetes, hypertension, and survey cycle

### Associations between urinary OPP metabolites and obesity and abdominal obesity

After adjusting for all variables, the OR (95% CI) of obesity in the multiple logistic regression analysis (Table 3) was 0.91 (0.87–0.94) for DMP, 0.94 (0.89–0.98) for DEP, 0.88 (0.85–0.92) for DMTP, and 0.81 (0.76–0.87) for DETP. Furthermore, DMP (OR [95% CI]: 0.93 [0.89–0.98]), DEP (OR [95% CI]: 0.94 [0.90–0.99]), DMTP (OR [95% CI]: 0.91 [0.86–0.95]) and DETP (OR [95% CI]: 0.85 [0.80–0.90]) were negatively associated with the prevalence of abdominal obesity after adjusting for confounders.

**Table 3:** Multiple logistic regression associations of urinary organophosphorus pesticide (OPP) metabolites with obesity and abdominal obesity in adults

Ops	Model	Obesity		Abdominal obesity	
		OR (95%CI)	*P* value	OR (95%CI)	*P* value
DMP	Model 1	0.93 (0.90–0.96)	< 0.001	0.99 (0.96–1.03)	0.780
	Model 2	0.91 (0.87–0.94)	< 0.001	0.92 (0.88–0.97)	< 0.001
	Model 3	0.91 (0.87–0.94)	< 0.001	0.93 (0.89–0.98)	0.003
DEP	Model 1	0.98 (0.94–1.03)	0.472	1.02 (0.98–1.06)	0.342
	Model 2	0.97 (0.92–1.01)	0.109	0.96 (0.92-1.00)	0.041
	Model 3	0.94 (0.89–0.98)	0.004	0.94 (0.90–0.99)	0.013
DMTP	Model 1	0.88 (0.85–0.92)	< 0.001	0.96 (0.92-1.00)	0.040
	Model 2	0.86 (0.83–0.90)	< 0.001	0.89 (0.85–0.93)	< 0.001
	Model 3	0.88 (0.85–0.92)	< 0.001	0.91 (0.86–0.95)	< 0.001
DETP	Model 1	0.82 (0.77–0.86)	< 0.001	0.90 (0.85–0.95)	< 0.001
	Model 2	0.80 (0.75–0.84)	< 0.001	0.84 (0.79–0.89)	< 0.001
	Model 3	0.81 (0.76–0.87)	< 0.001	0.85 (0.80–0.90)	< 0.001

BMI, body mass index; OR, odds ratio; CI, confidence interval

Model 1 was adjusted for urinary creatinine;

Model 2 was adjusted for age, sex, race, and urinary creatinine;

Model 3 was adjusted for age, sex, race, urinary creatinine, poverty, smoking status, drinking status, energy intake levels, HEI, physical activity, diabetes, hypertension, and survey cycle

### Effect of the mixture of urinary OPP metabolites on adiposity measures

**Fig. 1:**
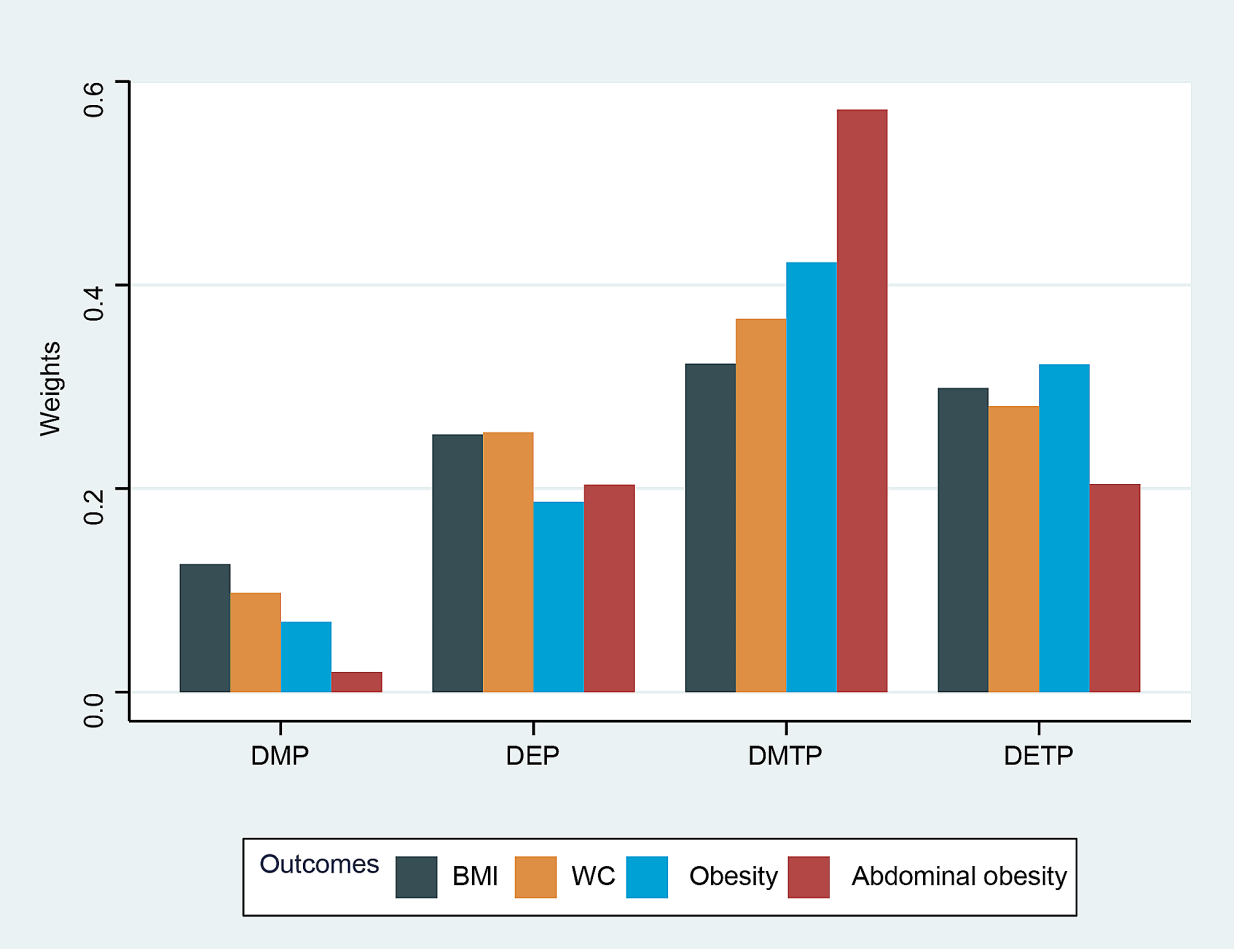
Weights from weighted quantile sum (WQS) regression for the mixture of urinary organophosphorus pesticide (OPP) metabolites and adiposity measures. Model was adjusted for adjusted as age, sex, race, urinary creatinine, poverty, smoking status, drinking status, energy intake levels, physical activity, diabetes, and hypertension

**Fig. 2:**
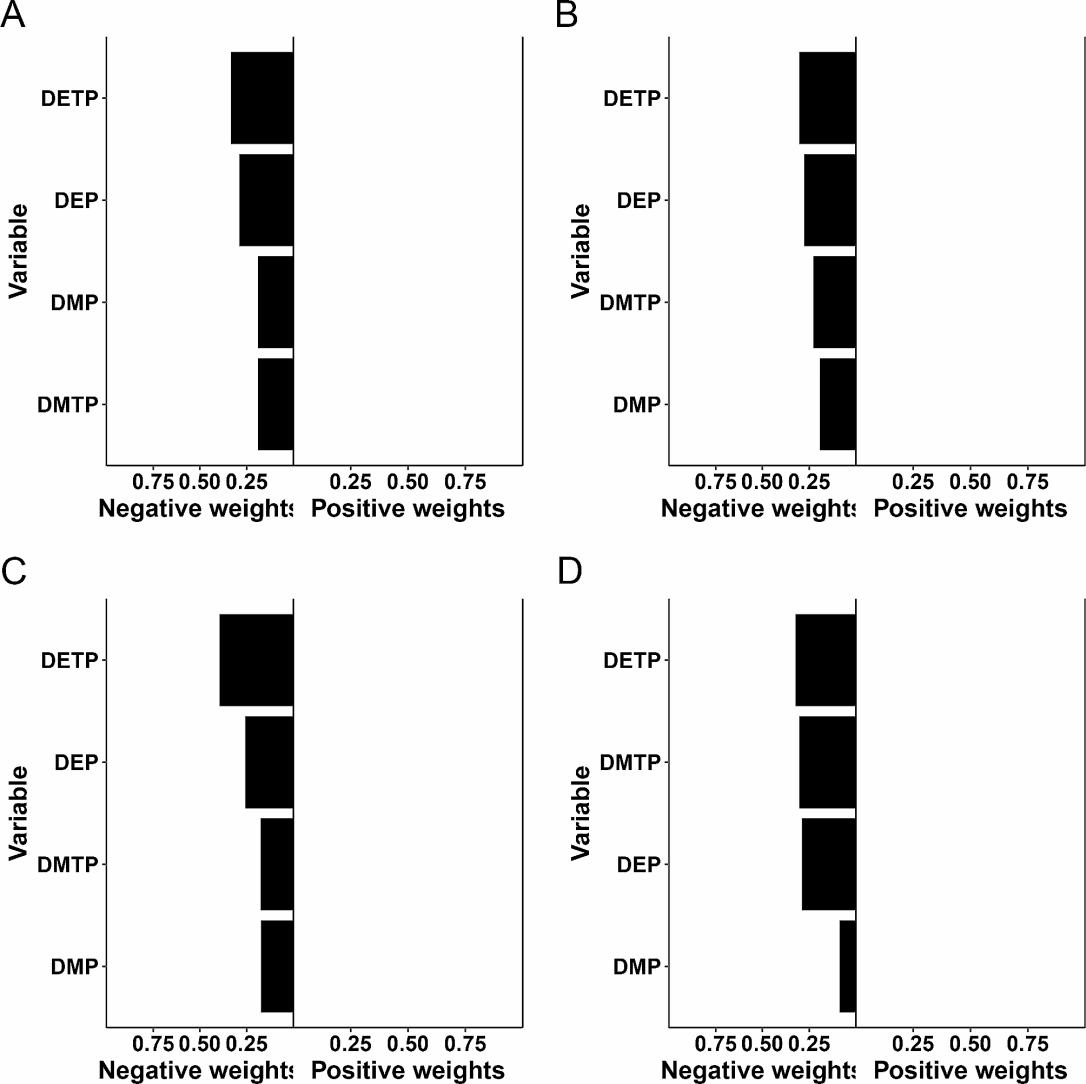
Weights from quantile g-computation (qgcomp) regression for the mixture of urinary organophosphorus pesticide (OPP) metabolites and adiposity measures (**A**: BMI, **B**: WC, **C**: Obesity, and **D**: Abdominal obesity). Model was adjusted for adjusted as age, sex, race, urinary creatinine, poverty, smoking status, drinking status, energy intake levels, physical activity, diabetes, and hypertension

The negative relationships between the mixture of urinary OPP metabolites and adiposity measures were analysed using WQS regression models (Table 4). The WQS index of the urinary OPP metabolites was independently associated with BMI (β [95% CI]: −0.29 [− 0.39, − 0.20]) and WC (β [95% CI]: −0.64 [− 0.86, − 0.42]). This model showed that the mixture of urinary OPP metabolites was associated with a decreased prevalence of obesity (OR [95% CI]: 0.93 [0.90–0.96]) and abdominal obesity (OR [95% CI]: 0.95 [0.92–0.98]). After adjusting for all covariates (Fig. 1), DMTP was the predominant OPPs that was negatively correlated with adiposity measures, with a weight of 0.32 for BMI, 0.37 for WC, 0.42 for obesity, and 0.57 for abdominal obesity. Additionally, the outcomes of the WQS regression analysis and the qgcomp regression analysis were in agreement (Table 5). Adiposity measures were separately and unfavourably correlated with the combination of urine OPP metabolites. The negative impact of mixed urine OPP metabolites on adiposity measures was primarily attributed to DETP according to the qgcomp analysis (weight = 0.334 for BMI, weight = 0.303 for WC, weight = 0.396 for obesity, and weight = 0.324 for abdominal obesity) (Fig. 2).

**Table 4:** WQS regression model to assess the negative association of the mixture of urinary organophosphorus pesticide (OPP) metabolites with adiposity measures

	OR / β	95%CI	*P* value
BMI	-0.29	-0.39, -0.20	< 0.001
Waist circumference	-0.64	-0.86, -0.42	< 0.001
Obesity	0.93	0.90–0.96	< 0.001
Abdominal obesity	0.95	0.92–0.98	< 0.001

OR: odds ratio; CI: confidence interval; BMI, body mass index; WQS: weighted quantile sum. WQS regression model was adjusted age, sex, race, urinary creatinine, poverty, smoking status, drinking status, energy intake levels, HEI, physical activity, diabetes, hypertension, and survey cycle

**Table 5:** Qgcomp regression to assess the negative association of the mixture of urinary organophosphorus pesticide (OPP) metabolites with adiposity measures

	OR / β	95%CI	*P* value
BMI	-0.30	-0.38, -0.23	< 0.001
Waist circumference	-0.73	-0.90, -0.55	< 0.001
Obesity	0.91	0.88–0.93	< 0.001
Abdominal obesity	0.92	0.89–0.94	< 0.001

OR: odds ratio; CI: confidence interval; BMI, body mass index; Qgcomp, quantile g-computation

Qgcomp regression model was adjusted as age, sex, race, urinary creatinine, poverty, smoking status, drinking status, energy intake levels, HEI, physical activity, diabetes, hypertension, and survey cycle

### Sensitivity analysis

To ensure the robustness of our findings, we conducted several sensitivity analyses by adjusting for additional covariates, including dietary nutrients, water consumption, depression symptoms, sleep duration, and urine dilution. After adjusting for dietary nutrients (including total dietary protein, carbohydrates, sugars, fiber, fat and cholesterol) and water consumption, the negative associations between urinary OPP metabolites and adiposity measures remained consistent and significant (Table S3). Similarly, adjustments for depression symptoms using Patient Health Questionnaire (PHQ) scores, both as continuous and categorical variables, did not alter the observed associations (Table S4). Including sleep duration, either as a continuous or categorized variable, also upheld the significant negative associations (Table S5). Finally, adjustments for urine dilution through urinary creatinine concentration, osmolality, and urinary flow rate continued to show robust negative associations between urinary OPP metabolites and adiposity measures (Table S6). These sensitivity analyses confirm the stability and reliability of the observed associations.

## Discussion

We investigated the relationship between four urinary OPP metabolites (DMP, DEP, DMTP, and DETP) and adiposity measures (BMI, WC, obesity, and abdominal obesity) in 9505 American adults using cross-sectional data from the 2003–2018 NHANES. A negative association was found between the urinary OPP metabolites (DMP, DMTP and DETP) and adiposity measures. According to the WQS regression model and the qgcomp regression model, the mixture of urinary OPP metabolites was negatively associated with adiposity measures, with DMTP and DETP showing the most significant effects.

Obesity is a complicated illness that involves too much body fat and raises the possibility of various major health problems. Besides excessive calorie intake and the lack of physical activity, exposure to chemical pollutants is considered a risk factor in the current obesity epidemic [15]. OPPs have endocrine-disrupting properties [33]. The blood malathion levels (a common OPPs used in agriculture) significantly increased along with increases in WC and BMI [34]. Adult farmers who are exposed to pesticides over an extended period of time have a higher prevalence of being overweight, but not abdominal obesity [21]. An extensive prevalence of obesity was strongly correlated with the use of eight OPPs, according to a population-based cross-sectional investigation [22]. To the best of our knowledge, this study is the first to extensively explore the relationship between urinary OPP metabolites and adiposity measures in an adult population. The urinary OPP metabolites and adiposity measures were shown to have statistically significant inverse correlations. More research is needed to confirm our findings and understand if an increase exposure to OPPs is inversely related to obesity in the adult population of American.

OPPs have been linked to obesity, and a recent comprehensive review of the literature concluded that most studies found the association between OP exposure and weight increase or decrease [33]. However, the potential mechanisms through which OPP metabolites might influence obesity-related outcomes warrant a more in-depth discussion. Some studies have reported weight loss in animals exposed to OPPs, such as dichlorvos, possibly due to a decrease in food intake [35]. Similarly, an experimental investigation found that both male and female rats exposed to an OP pesticide mixture had significantly lower mean body weights compared to the control group [36]. Exposure to OP pesticides has been associated with various adverse health effects that could indirectly influence body weight. For example, OPPs can cause neurofunctional impairments and sleep disturbances [37], which may reduce appetite and subsequently lead to weight loss. Additionally, gastrointestinal dysfunction caused by OPPs poisoning, including chlorpyrifos exposure, has been linked to lower body weight and length in pregnant rats. This could be due to disrupted nutrient absorption, gut microbial dysbiosis, and alterations in the mucosal barrier [38, 39]. These findings suggest that exposure to certain OPPs can lead to weight reduction, potentially through mechanisms such as decreased appetite, gastrointestinal dysfunction, and metabolic disturbances.

Another important consideration is the lipophilic properties of certain OPPs [40]. Although most OPPs do not significantly accumulate in fat, some, such as chlorpyrifos, diazinon, and parathion, exhibit moderate lipophilicity [41]. These compounds can temporarily reside in fatty tissues, meaning that individuals with higher body fat might have a greater capacity to retain OPPs, potentially leading to lower excretion values [42]. This could partly explain the negative correlation between urinary OPP metabolites and obesity, as individuals with more adipose tissue might sequester more of these lipophilic compounds, resulting in lower urinary concentrations. Prior to our analysis, animal experimental trials accounted for the majority of investigations on OPPs exposure and obesity. Population studies have not produced enough data to analyse this association, and the current study adds new epidemiological data. Pesticide exposure should be kept to a minimum due to the widespread use of OPPs and the diversity of health effects they cause. The current study therefore advises against the use of OPPs, particularly for those with low body weight.

The main strength is that the present study provided new epidemiological evidence to identify the relationships between OPP metabolites in urine and adiposity measures based on this large, representative population. Second, while earlier research concentrated on the relationships between exposure to a single OPP and outcomes associated to obesity, our study examined the effects of exposure to a variety of OPPs on outcomes linked to obesity. Finally, we made sure that the study’s most well-known confounding variables were adjusted.

There are a few restrictions on this study. Firstly, the cross-sectional nature of our study limits our ability to establish causality between urinary OPP metabolites and obesity measures. Cross-sectional studies provide a snapshot of data at a single point in time, which precludes the determination of temporal sequence between exposure and outcome. Therefore, while our findings demonstrate associations between urinary OPP metabolites and obesity measures, we acknowledge that these associations do not imply causation. Future studies should aim to overcome the limitations of cross-sectional designs by employing longitudinal or prospective cohort studies. Secondly, as urine OPP metabolites were only examined once and not repeatedly, the findings might not adequately depict the consequences of long-term exposure to OPPs. Additionally, our study is using the average of two 24-hour dietary recalls to estimate energy intake across multiple NHANES cycles. This method does not account for dietary changes over time, like adopting ketogenic diets or vegetarianism, which can affect weight and metabolism. Although we controlled for dietary intake in our models, more detailed and longitudinal dietary data would improve our findings. Thirdly, due to the poor detection rates of two OPP metabolites, we were unable to evaluate the combined impacts of all OPP metabolites using WQS regression and gqcomp regression.

Lastly, differences in the metabolism of individual OPPs may have influenced our results [43, 44]. It is important to consider that the observed associations between urinary OPP metabolites and obesity may be influenced by factors related to body weight and urinary excretion. Obese individuals often have higher muscle mass, leading to increased production and excretion of creatinine, which is commonly used to correct urine measurements. This could result in variations in metabolite levels that are independent of actual exposure. Additionally, obese individuals typically consume more fluids, potentially causing a dilution effect in urinary concentrations of OPP metabolites. This dilution could affect the measured values, making it appear as though there is a stronger or weaker association with obesity than truly exists. Furthermore, kidney function might be compromised, which can alter the excretion rates of various metabolites. Impaired kidney function can lead to either an accumulation or a faster clearance of metabolites, further complicating the interpretation of urinary biomarker data. In this study, our analysis model adjusted for urinary creatinine levels and water consumption to mitigate these confounding effects. By correcting for these factors, we aimed to reduce the influence of variations in muscle mass and fluid intake on the measurement of urinary OPP metabolites. Despite these adjustments, it remains crucial to acknowledge that other unmeasured factors related to obesity and urinary excretion could still impact our findings.

## Conclusion

Together, we discovered a link between urine OPP metabolites and outcomes connected to obesity in American people. According to our research, the combination of urinary OPP metabolites is linked to a lower prevalence of obesity, with DMTP and DETP showing the strongest associations.

### Electronic supplementary material

Below is the link to the electronic supplementary material.

Supplementary Material 1

## Data Availability

No datasets were generated or analysed during the current study.
