# Supplementary material for: Association between organophosphorus pesticides and obesity among American adults
Source: Environ Health. 2024 Jul 20;23:65. doi: 10.1186/s12940-024-01104-z (PMC11264883; doi:10.1186/s12940-024-01104-z)
Supplement: Supplementary file 1 — Supplementary Material 1 [file 12940_2024_1104_MOESM1_ESM.docx]

**Online Supplementary Material**

**Association between organophosphorus pesticides and obesity among American adults**

**Figure S1**. Eligible participants in the evaluation of the influence between urinary organophosphorus pesticide (OPP) metabolites and obesity-related outcomes in the general adult population.

**Table S1.** Concentrations and detection rate of the urinary organophosphorus pesticide (OPP) metabolites (ng/mL).

**Table S2.** Estimated weighted and geometric mean (GM) and 95% confidence interval (95%CI) of urinary organophosphorus pesticide (OPP) metabolites (in ng/mL) at each NHANES cycle.

**Figure S2.** Pairwise Spearman correlation coefficients among urinary organophosphorus pesticide (OPP) metabolites in the general population.

**Table S3.** Associations of urinary organophosphorus pesticide (OPP) metabolites with adiposity measures with further adjustment of dietary nutrients and water intake in adults.

**Table S4.** Associations of urinary organophosphorus pesticide (OPP) metabolites with adiposity measures with further adjustment of depression symptoms in adults.

**Table S5.** Associations of urinary organophosphorus pesticide (OPP) metabolites with adiposity measures with further adjustment of sleep duration in adults.

**Table S6.** Associations of urinary organophosphorus pesticide (OPP) metabolites with adiposity measures with further adjustment of urine dilution in adults.

**Materials and methods: Other Covariates**

This section provides a comprehensive definition of potential confounding variables including smoking status (never, former, or current smoker), drinking status (nondrinker, former drinker, or current drinker), physical activity (inactive, insufficiently active, or active), total energy intakes, and healthy eating index (HEI).

***Smoking status*** Never smokers were classified as those who reported smoking <100 cigarettes during their lifetime. Those who smoked >100 cigarettes in their lifetime were considered as current smokers, and those who smoked >100 cigarettes and had quit smoking were considered as former smokers [1].

***Drinking status*** Drinking status was classified as nondrinker, low-to-moderate drinker (<2 drinks/day in men and <1 drink/day in women), or heavy drinker (≥2 drinks/day in men and ≥1 drinks/day in women) [1].

***Physical activity*** Physical activity was categorized as inactive group (no leisure-time physical activity), insufficiently active group (leisure time moderate activity 1–5 times per week with MET ranging from 3 to 6 or leisure-time vigorous activity 1–3 times per week with MET >6), or active group (those who had more leisure-time moderate-or-vigorous activity than above) [2].

***Healthy Eating Index*** The Healthy Eating Index (HEI) is a measure calculated from 24-hour dietary recall data to assess diet quality based on the 2015–2020 Dietary Guidelines for Americans (DGA) [3]. It comprises 13 subgroups, with a total possible score of 100. Nine components evaluate adequacy (higher intakes contribute to a higher score) including total fruits, whole fruits, total vegetables, greens and beans, whole grains, dairy, total protein foods, seafood and plant proteins, and fatty acids. The remaining four components assess moderation (lower intakes yield a higher score), covering refined grains, sodium, added sugars, and saturated fats. The HEI offers a comprehensive framework for understanding dietary patterns and their relationship to health outcomes, with scores reflecting adherence to key dietary recommendations.

***Total dietary energy intakes*** Dietary intake information was collected using two 24-hour dietary recall interviews conducted as part of the National Health and Nutrition Examination Survey (NHANES). The first dietary recall interview was administered in-person at the NHANES Mobile Examination Center (MEC), while the second recall was conducted via telephone 3 to 10 days later. Participants were asked to recall all foods and beverages consumed during the previous 24-hour period (midnight to midnight) for each recall day. The total energy intake was calculated using the USDA's Automated Multiple-Pass Method (AMPM). The Food and Nutrient Database for Dietary Studies (FNDDS) was used to analyze the reported dietary intake data and estimate total energy intake in kilocalorie. The average energy intake from the two 24-hour dietary recalls was used for our analysis. Detailed information on the NHANES Dietary Analysis can be found at: <https://wwwn.cdc.gov/nchs/nhanes/tutorials/dietaryanalyses.aspx>.

**References**

1. Qiu Z, Chen X, Geng T, Wan Z, Lu Q, Li L, Zhu K, Zhang X, Liu Y, Lin X *et al*: **Associations of Serum Carotenoids With Risk of Cardiovascular Mortality Among Individuals With Type 2 Diabetes: Results From NHANES**. *Diabetes Care* 2022, **45**(6):1453-1461.

2. Beddhu S, Baird BC, Zitterkoph J, Neilson J, Greene T: **Physical activity and mortality in chronic kidney disease (NHANES III)**. *Clin J Am Soc Nephrol* 2009, **4**(12):1901-1906.

3. Kirkpatrick SI, Reedy J, Krebs-Smith SM, Pannucci TE, Subar AF, Wilson MM, Lerman JL, Tooze JA: **Applications of the Healthy Eating Index for Surveillance, Epidemiology, and Intervention Research: Considerations and Caveats**. *J Acad Nutr Diet* 2018, **118**(9):1603-1621.

**

**

**Figure S1**. Eligible participants in the evaluation of the influence between urinary organophosphorus pesticide (OPP) metabolites and obesity-related outcomes in the general adult population.

**Table S1**. Concentrations and detection rate of the urinary organophosphorus pesticide (OPP) metabolites (ng/mL).

| Urinary OPP metabolites | Common Name | N | ≥LODmax^a^ | 25^th^ | 50^th^ | 75^th^ |
| --- | --- | --- | --- | --- | --- | --- |
| Dimethylphosphate | DMP | 15922 | 71.1% | 0.350 | 1.250 | 4.260 |
| Diethylphosphate | DEP | 15923 | 69.9% | 0.262 | 1.390 | 4.250 |
| Dimethylthiophosphate | DMTP | 15950 | 83.7% | 0.390 | 1.260 | 3.970 |
| Diethylthiophosphate | DETP | 15855 | 52.5% | 0.140 | 0.396 | 0.646 |
| Dimethyldithiophosphate | DMDTP | 15900 | 40.6% | 0.070 | 0.325 | 0.361 |
| Diethyldithiophosphate | DEDTP | 15743 | 3.5% | 0.070 | 0.070 | 0.276 |

N, number of urinary samples; LOD, limit of detection; 25^th^, 25th percentile; 50^th^, 50th percentile; 75^th^, 75th percentile.

^a^ Percentage of metabolite concentrations at or above the maximum limit of detection (< LODmax). All concentrations below the LODmax (< LODmax) were substituted with a value of LODmax divided by square root of two (√2).

**Table S2.** Estimated weighted and geometric mean (GM) and 95% confidence interval (95%CI) of urinary organophosphorus pesticide (OPP) metabolites (in ng/mL) at each NHANES cycle.

| Urinary OP metabolites | All NHANES Cycles | 2003-2004 | 2005-2006 | 2007-2008 | 2011-2012 | 2015-2016 | 2017-2018 | *P*_trend_ |
| --- | --- | --- | --- | --- | --- | --- | --- | --- |
| DMP | 1.38(1.30,1.46) | 1.27(1.06,1.53) | 1.06(0.91,1.24) | 1.26(1.10,1.44) | 2.32(2.07,2.61) | 1.37(1.24,1.51) | 1.24(1.09,1.41) | <0.001 |
| DEP | 1.17(1.08,1.27) | 0.56(0.38,0.83) | 0.59(0.53,0.66) | 0.66(0.59,0.74) | 2.14(1.90,2.40) | 1.99(1.77,2.23) | 2.13(1.96,2.31) | <0.001 |
| DMTP | 1.34(1.26,1.42) | 2.31(2.05,2.60) | 1.82(1.64,2.04) | 2.15(1.80,2.57) | 1.59(1.38,1.84) | 0.74(0.65,0.84) | 0.67(0.55,0.81) | <0.001 |
| DETP | 0.32(0.31,0.34) | 0.36(0.32,0.41) | 0.56(0.53,0.59) | 0.67(0.63,0.73) | 0.29(0.25,0.33) | 0.21(0.18,0.23) | 0.17(0.16,0.19) | <0.001 |
| DMDTP | 0.25(0.24,0.27) | 0.22(0.18,0.26) | 0.51(0.47,0.56) | 0.55(0.51,0.59) | 0.21(0.19,0.24) | 0.15(0.14,0.17) | 0.15(0.13,0.17) | <0.001 |
| DEDTP | 0.11(0.11,0.12) | 0.08(0.08,0.08) | 0.28(0.28,0.29) | 0.28(0.28,0.28) | 0.07(0.07,0.07) | 0.07(0.07,0.07) | 0.07(0.07,0.07) | <0.001 |


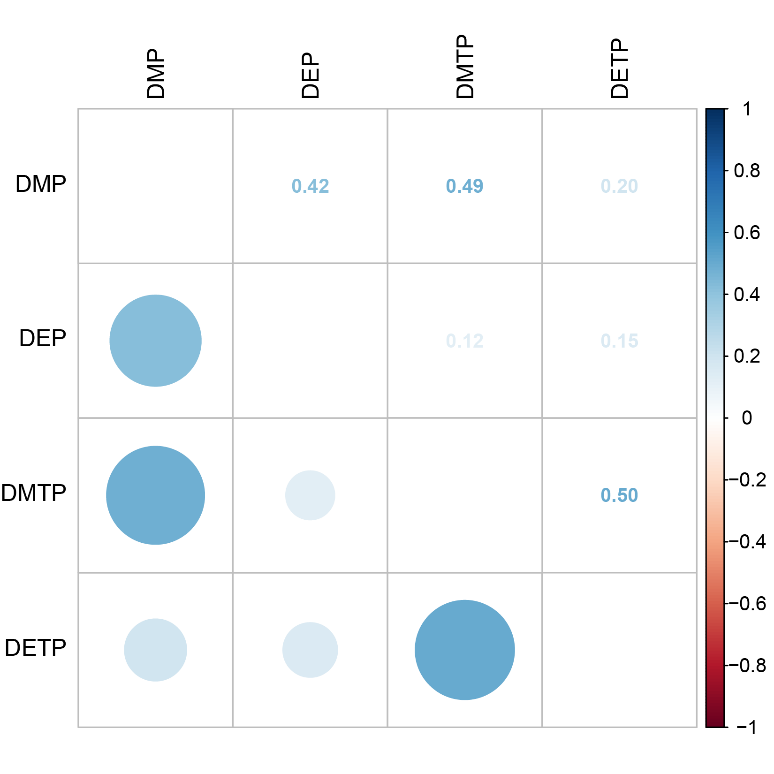


**Figure S2**. Pairwise Spearman correlation coefficients among urinary organophosphorus pesticide (OPP) metabolites in the general population.

**Table S3.** Associations of urinary organophosphorus pesticide (OPP) metabolites with adiposity measures with further adjustment of dietary nutrients and water intake in adults.

| OPs | BMI | |  | Waist circumference | |  | Obesity | |  | Abdominal obesity | |
| --- | --- | --- | --- | --- | --- | --- | --- | --- | --- | --- | --- |
|  | β (95% CI) | *P* value |  | β (95% CI) | *P* value |  | OR (95% CI) | *P* value |  | OR (95% CI) | *P* value |
| Model + Dietary nutrients * | | |  |  |  |  |  |  |  |  |  |
| DMP | -0.26(-0.38, -0.15) | <0.001 |  | -0.67(-0.96, -0.37) | <0.001 |  | 0.92(0.89-0.95) | <0.001 |  | 0.94(0.89-0.99) | 0.012 |
| DEP | -0.21(-0.32, -0.09) | <0.001 |  | -0.52(-0.78, -0.26) | <0.001 |  | 0.94(0.90-0.98) | 0.005 |  | 0.94(0.90-0.99) | 0.016 |
| DMTP | -0.33(-0.44, -0.21) | <0.001 |  | -0.8(-1.08, -0.52) | <0.001 |  | 0.90(0.86-0.94) | <0.001 |  | 0.92(0.87-0.96) | <0.001 |
| DETP | -0.55(-0.71, -0.39) | <0.001 |  | -1.24(-1.63, -0.85) | <0.001 |  | 0.82(0.77-0.88) | <0.001 |  | 0.86(0.81-0.91) | <0.001 |
| Model + Water intake | | |  |  |  |  |  |  |  |  |  |
| DMP | -0.30(-0.41, -0.18) | <0.001 |  | -0.75(-1.04, -0.46) | <0.001 |  | 0.91(0.87-0.94) | <0.001 |  | 0.93(0.89-0.97) | 0.003 |
| DEP | -0.21(-0.33, -0.10) | <0.001 |  | -0.54(-0.80, -0.27) | <0.001 |  | 0.94(0.89-0.98) | 0.005 |  | 0.94(0.90-0.99) | 0.013 |
| DMTP | -0.38(-0.49, -0.27) | <0.001 |  | -0.93(-1.19, -0.66) | <0.001 |  | 0.88(0.85-0.92) | <0.001 |  | 0.90(0.86-0.95) | <0.001 |
| DETP | -0.59(-0.75, -0.43) | <0.001 |  | -1.34(-1.72, -0.95) | <0.001 |  | 0.81(0.76-0.87) | <0.001 |  | 0.85(0.80-0.90) | <0.001 |

Model was adjusted for age, sex, race, urinary creatinine, poverty, smoking status, drinking status, energy intake levels, HEI, physical activity, diabetes, hypertension, and survey cycle.

* Dietary nutrients include total dietary protein, carbohydrates, sugars, fiber, fat and cholesterol (all ln-transformed).

**Table S4.** Associations of urinary organophosphorus pesticide (OPP) metabolites with adiposity measures with further adjustment of depression symptoms in adults.

| OPs | BMI | |  | Waist circumference | |  | Obesity | |  | Abdominal obesity | |
| --- | --- | --- | --- | --- | --- | --- | --- | --- | --- | --- | --- |
|  | β (95% CI) | *P* value |  | β (95% CI) | *P* value |  | OR (95% CI) | *P* value |  | OR (95% CI) | *P* value |
| Model + PHQ-9 scores (continous) * | | |  |  |  |  |  |  |  |  |  |
| DMP | -0.32(-0.46, -0.19) | <0.001 |  | -0.79(-1.13, -0.45) | <0.001 |  | 0.90(0.86-0.94) | <0.001 |  | 0.92(0.88-0.97) | 0.003 |
| DEP | -0.26(-0.40, -0.11) | <0.001 |  | -0.62(-0.95, -0.30) | <0.001 |  | 0.93(0.88-0.98) | 0.010 |  | 0.93(0.88-0.99) | 0.023 |
| DMTP | -0.37(-0.49, -0.25) | <0.001 |  | -0.90(-1.21, -0.59) | <0.001 |  | 0.88(0.84-0.92) | <0.001 |  | 0.89(0.85-0.94) | <0.001 |
| DETP | -0.67(-0.85, -0.49) | <0.001 |  | -1.55(-2.00, -1.10) | <0.001 |  | 0.79(0.73-0.85) | <0.001 |  | 0.82(0.77-0.88) | <0.001 |
| Model + Depression (PHQ-9 scores ≥ 10) * | | | |  |  |  |  |  |  |  |  |
| DMP | -0.32(-0.45, -0.19) | <0.001 |  | -0.78(-1.12, -0.44) | <0.001 |  | 0.90(0.86-0.94) | <0.001 |  | 0.92(0.88-0.97) | 0.004 |
| DEP | -0.26(-0.40, -0.12) | <0.001 |  | -0.63(-0.96, -0.30) | <0.001 |  | 0.93(0.88-0.98) | 0.010 |  | 0.93(0.88-0.99) | 0.023 |
| DMTP | -0.37(-0.49, -0.25) | <0.001 |  | -0.9(-1.21, -0.59) | <0.001 |  | 0.88(0.84-0.92) | <0.001 |  | 0.90(0.85-0.95) | <0.001 |
| DETP | -0.68(-0.86, -0.50) | <0.001 |  | -1.57(-2.01, -1.12) | <0.001 |  | 0.79(0.73-0.85) | <0.001 |  | 0.82(0.77-0.88) | <0.001 |

Model was adjusted for age, sex, race, urinary creatinine, poverty, smoking status, drinking status, energy intake levels, HEI, physical activity, diabetes, hypertension, and survey cycle.

* The Patient Health Questionnaire (PHQ) scale, a nine-item depression screening instrument, was used to assess depression symptoms over the past two weeks. Each symptom is scored from 0 to 3, with total scores of 10 or more indicating depression. The PHQ scale was collected only in the NHANES 2005-2018.

**Table S5.** Associations of urinary organophosphorus pesticide (OPP) metabolites with adiposity measures with further adjustment of sleep duration in adults.

| OPs | BMI | |  | Waist circumference | |  | Obesity | |  | Abdominal obesity | |
| --- | --- | --- | --- | --- | --- | --- | --- | --- | --- | --- | --- |
|  | β (95% CI) | *P* value |  | β (95% CI) | *P* value |  | OR (95% CI) | *P* value |  | OR (95% CI) | *P* value |
| Model + Sleep duration (continous) * | | |  |  |  |  |  |  |  |  |  |
| DMP | -0.31(-0.44, -0.18) | <0.001 |  | -0.76(-1.10, -0.42) | <0.001 |  | 0.90(0.87-0.94) | <0.001 |  | 0.93(0.88-0.98) | 0.005 |
| DEP | -0.25(-0.39, -0.11) | <0.001 |  | -0.61(-0.94, -0.28) | <0.001 |  | 0.93(0.88-0.98) | 0.012 |  | 0.93(0.88-0.99) | 0.027 |
| DMTP | -0.36(-0.48, -0.23) | <0.001 |  | -0.87(-1.18, -0.56) | <0.001 |  | 0.88(0.84-0.92) | <0.001 |  | 0.90(0.85-0.95) | <0.001 |
| DETP | -0.67(-0.85, -0.49) | <0.001 |  | -1.55(-2.00, -1.10) | <0.001 |  | 0.79(0.73-0.85) | <0.001 |  | 0.82(0.77-0.88) | <0.001 |
| Model + Sleep duration (categorized) * | | |  |  |  |  |  |  |  |  |  |
| DMP | -0.31(-0.44, -0.18) | <0.001 |  | -0.76(-1.10, -0.42) | <0.001 |  | 0.90(0.87-0.94) | <0.001 |  | 0.93(0.88-0.98) | 0.005 |
| DEP | -0.25(-0.39, -0.11) | <0.001 |  | -0.61(-0.94, -0.28) | <0.001 |  | 0.93(0.88-0.98) | 0.013 |  | 0.93(0.88-0.99) | 0.027 |
| DMTP | -0.36(-0.48, -0.24) | <0.001 |  | -0.88(-1.19, -0.56) | <0.001 |  | 0.88(0.84-0.92) | <0.001 |  | 0.90(0.85-0.95) | <0.001 |
| DETP | -0.67(-0.85, -0.49) | <0.001 |  | -1.56(-2.02, -1.10) | <0.001 |  | 0.79(0.73-0.85) | <0.001 |  | 0.82(0.77-0.88) | <0.001 |

Model was adjusted for age, sex, race, urinary creatinine, poverty, smoking status, drinking status, energy intake levels, HEI, physical activity, diabetes, hypertension, and survey cycle.

* Sleep duration was self-reported and assessed by asking participants the average number of hours they sleep at night on workdays. Consistent with previous studies, sleep duration was categorized as short (≤ 6 hours), recommended (> 6 to < 9 hours), and long (≥ 9 hours). Sleep duration data were collected only in the NHANES 2005-2018.

**Table S6.** Associations of urinary organophosphorus pesticide (OPP) metabolites with adiposity measures with further adjustment of urine dilution in adults.

| OPs | BMI | |  | Waist circumference | |  | Obesity | |  | Abdominal obesity | |
| --- | --- | --- | --- | --- | --- | --- | --- | --- | --- | --- | --- |
|  | β (95% CI) | *P* value |  | β (95% CI) | *P* value |  | OR (95% CI) | *P* value |  | OR (95% CI) | *P* value |
| Model 1 | | |  |  |  |  |  |  |  |  |  |
| DMP | -1.84(-3.22, -0.46) | 0.010 |  | -4.98(-8.29, -1.68) | 0.004 |  | 0.70(0.33-1.49) | 0.350 |  | 0.74(0.44-1.26) | 0.260 |
| DEP | -3.51(-5.83, -1.18) | 0.004 |  | -7.56(-13.37, -1.76) | 0.011 |  | 0.25(0.07-0.93) | 0.039 |  | 0.34(0.12-0.97) | 0.044 |
| DMTP | -1.32(-2.10, -0.53) | <0.001 |  | -3.26(-5.01, -1.50) | <0.001 |  | 0.51(0.28-0.93) | 0.031 |  | 0.66(0.46-0.94) | 0.022 |
| DETP | -13.52(-18.34, -8.69) | <0.001 |  | -29.08(-40.95, -17.21) | <0.001 |  | 0.00(0.00-0.36) | 0.021 |  | 0.01(0.00-0.19) | 0.003 |
| Model 2 | | |  |  |  |  |  |  |  |  |  |
| DMP | -0.31( -0.44, -0.18) | <0.001 |  | -0.75(-1.09, -0.41) | <0.001 |  | 0.90(0.87- 0.94) | <0.001 |  | 0.93(0.88- 0.98) | 0.005 |
| DEP | -0.25( -0.39, -0.11) | <0.001 |  | -0.61(-0.93, -0.28) | <0.001 |  | 0.93(0.88- 0.98) | 0.013 |  | 0.93(0.88- 0.99) | 0.027 |
| DMTP | -0.36( -0.48, -0.24) | <0.001 |  | -0.87(-1.18, -0.56) | <0.001 |  | 0.88(0.84- 0.92) | <0.001 |  | 0.90(0.85- 0.95) | <0.001 |
| DETP | -0.67( -0.85, -0.49) | <0.001 |  | -1.56(-2.02, -1.10) | <0.001 |  | 0.79(0.73- 0.85) | <0.001 |  | 0.82(0.77- 0.88) | <0.001 |
| Model 3 | |  |  |  |  |  |  |  |  |  |  |
| DMP | -0.66(-0.91, -0.41) | 0.032 |  | -1.51(-2.20, -0.83) | <0.001 |  | 0.80(0.73-0.87) | <0.001 |  | 0.86(0.76-0.97) | 0.019 |
| DEP | -0.46(-0.76, -0.17) | 0.004 |  | -0.92(-1.56, -0.28) | 0.007 |  | 0.85(0.77-0.95) | 0.006 |  | 0.89(0.80-0.99) | 0.029 |
| DMTP | -0.46(-0.64, -0.28) | <0.001 |  | -1.12(-1.56, -0.68) | <0.001 |  | 0.85(0.80-0.91) | <0.001 |  | 0.87(0.80-0.95) | 0.003 |
| DETP | -0.69(-0.91, -0.46) | <0.001 |  | -1.54(-2.09, -0.98) | <0.001 |  | 0.78(0.72-0.85) | <0.001 |  | 0.83(0.77-0.90) | <0.001 |

Models refer to: 1) urinary OPs metabolites divided by urinary creatinine concentration (μg/g); 2) osmolality as an independent covariate in the regression model; 3) urinary flow rate as an independent covariate in the regression model. All models were additionally adjusted for age, sex, race, urinary creatinine, poverty, smoking status, drinking status, energy intake levels, HEI, physical activity, diabetes, hypertension, and survey cycle. Urinary flow rate data were collected only in the NHANES 2011-2018.
